# Artificial intelligence for intraoperative video analysis in robotic-assisted esophagectomy

**DOI:** 10.1007/s00464-025-11685-6

**Published:** 2025-03-31

**Authors:** Amila Cizmic, Anuja T. Mitra, Anas A. Preukschas, Marius Kemper, Nathaniel T. Melling, Oliver Mann, Sheraz Markar, Thilo Hackert, Felix Nickel

**Affiliations:** 1 Department of General, Visceral and Thoracic Surgery, University Medical Center Hamburg-Eppendorf, Martinistraße 52, 20246 Hamburg, Germany; 2 Department of Surgery & Cancer, Imperial College, London, UK; 3 Nuffield Department of Surgical Sciences, University of Oxford, Oxford, UK

**Keywords:** Artificial intelligence, Robotic-assisted esophagectomy, Minimally invasive surgery, Intraoperative video analysis

## Abstract

**Background:**

Robotic-assisted minimally invasive esophagectomy (RAMIE) is a complex surgical procedure for treating esophageal cancer. Artificial intelligence (AI) is an uprising technology with increasing applications in the surgical field. This scoping review aimed to assess the current AI applications in RAMIE, with a focus on intraoperative video analysis.

**Methods:**

To identify all articles utilizing AI in RAMIE, a comprehensive literature search was performed in accordance with the Preferred Reporting Items for Systematic Reviews and Meta-analysis for scoping reviews of Medline and Embase databases and the Cochrane Library. Two independent reviewers assessed articles for quality and inclusion.

**Results:**

One hundred and seventeen articles were identified, of which four were included in the final analysis. Results demonstrated that the main AI applications in RAMIE were intraoperative video assessment and the evaluation of surgical technical skills to evaluate surgical performance. AI was also used for surgical phase recognition to support clinical decision-making through intraoperative guidance and identify key anatomical landmarks. Various deep-learning networks were used to generate AI models, and there was a strong emphasis on using high-quality standardized video frames.

**Conclusions:**

The use of AI in RAMIE, especially in intraoperative video analysis and surgical phase recognition, is still a relatively new field that should be further explored. The advantages of using AI algorithms to evaluate intraoperative videos in an automated manner may be harnessed to improve technical performance and intraoperative decision-making, achieve a higher quality of surgery, and improve postoperative outcomes.

**Graphical Abstract:**

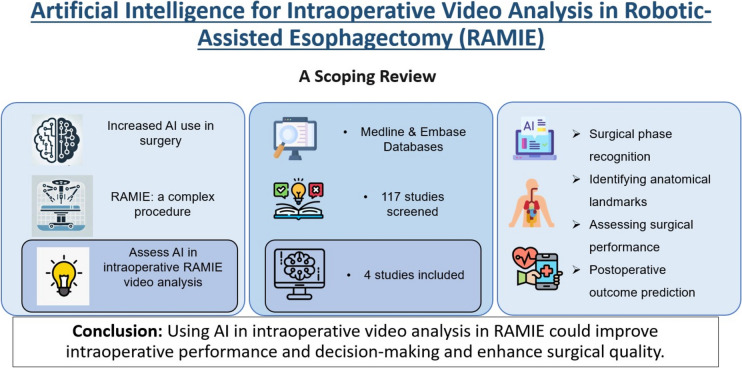

**Supplementary Information:**

The online version contains supplementary material available at 10.1007/s00464-025-11685-6.

One of the most aggressive gastrointestinal cancers is esophageal cancer (EC) [1]. EC is the world’s sixth most common cause of cancer death, with 600,000 new cases diagnosed annually [2, 3]. Surgical resection via esophagectomy with en bloc lymph node dissection is the cornerstone of curative treatment for locally advanced EC [4]. However, performing esophagectomy, regardless of the surgical approach, is a complex, technically challenging procedure linked to significant postoperative morbidity and mortality [5–8]. Robotic-assisted technology in general surgery is dramatically increasing as it allows more surgeons to offer their patients minimally invasive surgery (MIS). Robotic-assisted minimally invasive esophagectomy (RAMIE) has been demonstrated to be a safe and feasible surgical approach for treating patients with EC, generating acceptable postoperative outcomes such as length of hospital stay and equivocal oncological outcomes compared to open or conventional minimally invasive esophagectomy (MIE) [8–10]. RAMIE has been reported to allow a broader range of motion, superior optics, and more comfortable positioning and operating ergonomics for the surgeon [11, 12]. In addition, the technical development of the systems with advanced instrumentation has favored their use in complex visceral and thoracic surgical procedures [5]. As with MIE, RAMIE has a significant advantage of acquiring full-length intraoperative videos compared to open surgery. Analyzing these videos, including surgical phase recognition, image-guided surgery, and surgical performance assessment, could improve patient outcomes following esophagectomy.

Artificial intelligence (AI) is an extension of computer science that permits computers to build mathematical models to solve problems by mimicking human cognition, provided these models have been trained properly [13, 14]. A part of AI is machine learning (ML), a subdomain of AI roughly described as an automated extraction of knowledge from specific data [15]. Deep learning (DL), as a subset of ML, is usually described as a neural network with three or more layers of algorithms. These neural networks simulate the behavior of the human nervous system, allowing them to “learn” from data more independently [16]. In many medical applications, DL is often used in image recognition and video assessment [17–20]. Some DL models are large language models (LLMs). LLMs create new combinations of text that mimic the natural language based on its trained dataset. Generative AI uses LLMs as one of the models to create content like images, text, and synthetic data, among others. Preoperative planning and decision support, intraoperative documentation, and postoperative follow-up are just some of the potential applications of generative AI [21].

One AI field that has flourished exceptionally in various medical applications over the last couple of years is computer vision (CV). CV explores how computers can develop a high-level understanding of digital images or videos and perform functions such as object identification, object tracking, and scene recognition, which can be used in intraoperative video assessment (Fig. 1) [14, 22, 23]. The number of scientific publications on AI medical applications has increased rapidly over the past decade [24, 25]. Several AI algorithms have implemented CV in intraoperative video analyses and surgical phase recognition [26–32].

**Fig. 1:**
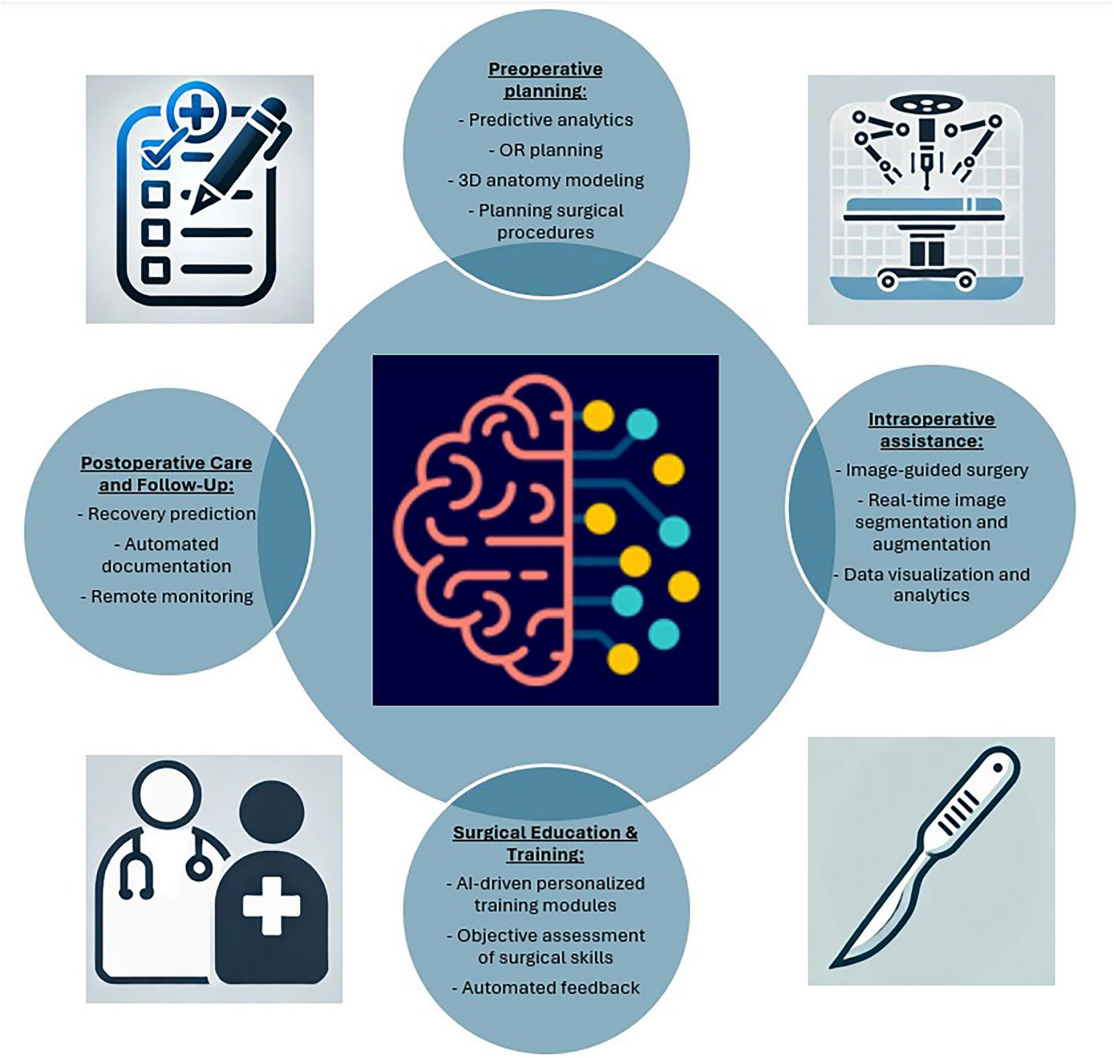
A brief overview of possible AI applications in surgery

RAMIE may benefit from AI as it is a lengthy, technically challenging procedure requiring operating in multiple anatomical compartments in the proximity of anatomical vital risk structures. In particular, the difficulty of accessing the upper thoracic compartment benefits from the superior views and broader range of motions that the RAMIE offers. Intraoperative applications of AI in RAMIE could offer image guidance, support in decision-making, personalized surgical management, and surgical training. This scoping review aimed to summarize the most current AI applications in intraoperative video analysis for RAMIE.

## Methods

**Fig. 2:**
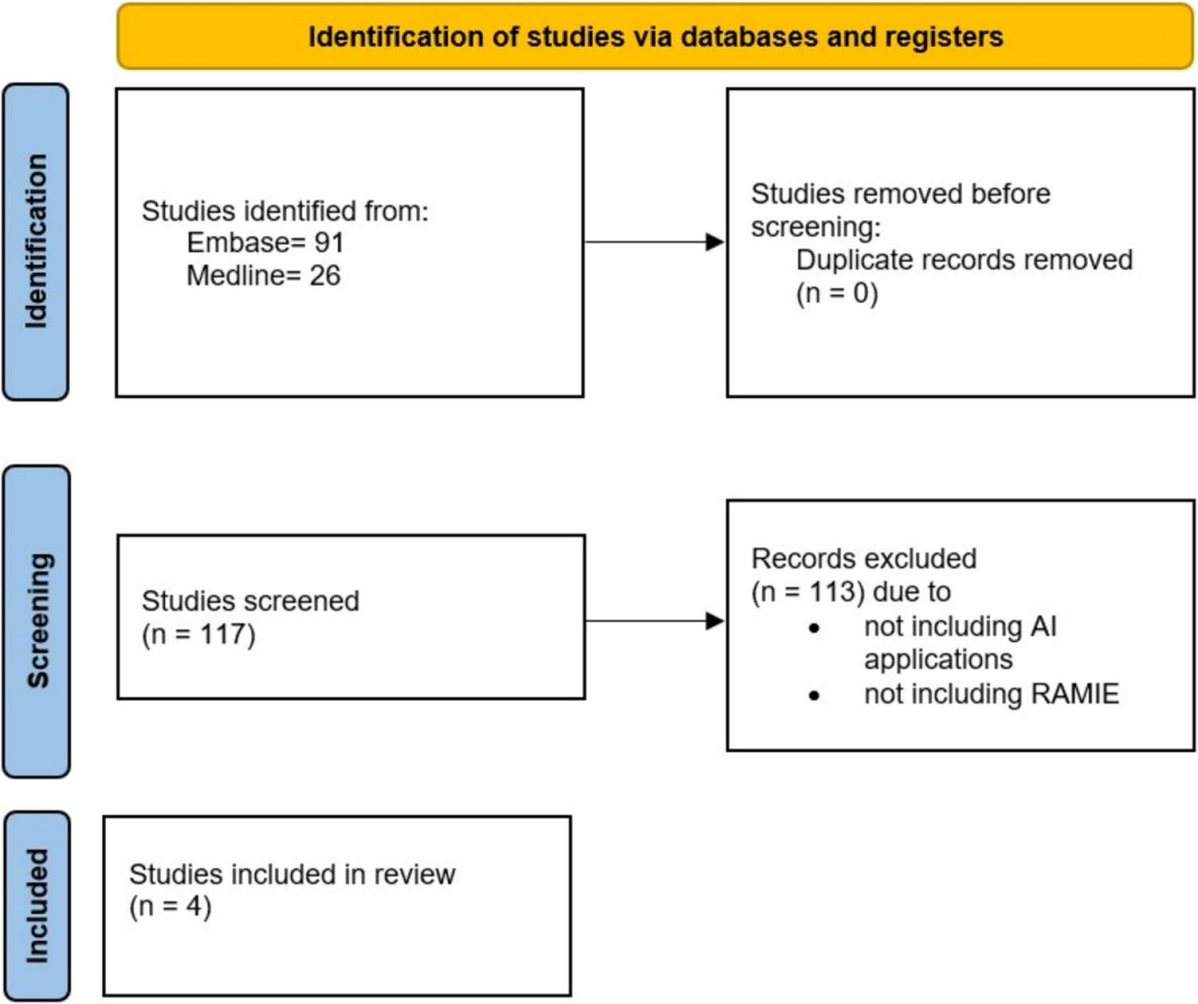
Flowchart of the study

This scoping review was performed in accordance with Preferred Reporting Items for Systematic Reviews and Meta-Analyses Extension for Scoping Reviews (PRISMA-ScR) to identify full-text publications reporting the use of AI in intraoperative video analysis in RAMIE. The databases searched included Medline (1946 to August 14, 2024) and Embase (1947 to August 14, 2024) via the Ovid platform, the Cochrane Review Library, and Scopus databases (Fig. 2). The reference lists of eligible articles were also hand-searched for additional publications. The entire search strategy can be found in Supplementary File 1. All variations in the spelling, including truncated search terms using wild card characters and the “related articles” function, were combined with the Boolean operators AND, OR. Reference lists of qualified articles were screened by two independent reviewers (A.C. and A.M.).

### Inclusion and exclusion criteria

Studies were included if they reported using AI in any aspect of RAMIE. All types of studies were considered for inclusion. The studies were excluded if they did not report on AI applications in RAMIE. The data extracted included the year of publication, study design, sample size, country of study, number of videos, number of participants, number of annotated frames, cross-fold validation techniques, and AI model details. All primary studies were appraised for quality and rigorousness using the STARD checklist, a 30-item essential checklist rating the quality and transparency of studies [33]. The STARD-AI checklist, once available for use, would be the most appropriate for this scoping review as it specifically addresses the issues and challenges raised by AI-centered interventions [34].

## Results

Out of 117 articles identified, four (*n* = 4) were included in the final analysis. Hundred and thirteen studies were excluded due to failing to report on AI applications, including ML, DL, and/or LLM models as well as not reporting on RAMIE. Table 1 summarizes the study characteristics. The included studies were published between 2022 and 2023. All studies were retrospective single-center reviews of cases. There were no randomized controlled trials.

**Table 1:** Summary table of included studies

Author	Takeuchi et al	De Backer et al	Den Boer et al	Brandenburg et al
Publication Year	2022	2022	2023	2023
Country	Japan	Belgium	Netherlands	Germany
Operation	RAMIE	RAPN and RAMIE	RAMIE	RAMIE
AI application	Surgical phase recognition	Requirements and methods for video instrument annotation	Anatomical landmark	Prediction of surgical outcomes based on annotation of surgical videos
Number of videos	31	82 RAPN and 15 RAMIE	83	22
Number of frames (av.)	14,515 ± 477	1232 (RAMIE)	1050	14,004
Networks used	Deep learning, TeCNO	Machine learning	Deep learning, CNN	Deep learning, CNN (Bayesian ResNet18)
Performance metrics/Outcome measures	Normalized confusion matrices, precision, recall, f value, and accuracy	Intersection over union (IoU), sampling period; time per frame for different annotation modalities	Dice and Hausdorff distances (primary) and pixel-wise accuracy, sensitivity, and specificity (secondary)	F1 score, precision, recall
Cross Validation	4	n/a	5	10
STARD checklist	22	25	23	26

*AI* artificial intelligence, *av* Average, *STARD* Standards for Reporting Diagnostic Accuracy Studies, *RAMIE* Robotic-assisted minimally invasive esophagectomy, *TeCNO* Temporal convolutional networks for the operating room, *RAPN* Robotic-assisted partial nephrectomy, *n/a* Not applicable, *CNN* Convolutional neural network

### Intraoperative video analysis

Esophagectomy is predominantly performed with a laparoscopic or robotic-assisted surgical approach, allowing surgeons to obtain, store, and assess video-recorded surgical procedures [35]. Integrating AI in the process of intraoperative video analysis of surgical procedures is ideal as it has the potential to automate and process vast quantities of video data efficiently, something that is not possible manually [36]. Some of intraoperative video analysis’s most important clinical applications are surgical phase recognition, surgical quality assessment, and intraoperative guidance.

### Surgical phase recognition

Takeuchi et al. established an AI model for automated surgical phase recognition in RAMIE [37]. The authors divided RAMIE into nine surgical phases and used an AI model known as Temporal Convolutional Networks for the Operating room (TeCNO), which utilized a temporal convolutional network for hierarchical prediction of surgical phases [38]. The AI performance was assessed by comparing the predictions of the AI model with the annotations of the surgical phases made by a surgeon. The overall accuracy of the AI model compared to the surgeon’s annotations was 84%, showing the acceptable effectiveness of the developed AI model for automated phase recognition for RAMIE.

Brandenburg et al. sought to develop an active learning (AL) ML program to annotate robotic intraoperative videos to reduce the manual annotation effort [39]. The AL program aimed to identify video frames significant for surgical bleeding as an indicator of overall patient outcomes from 22 RAMIE videos. The overarching goal was to utilize intraoperative videos guided by frame selection from AL to improve surgical therapy by extracting quantitative information from surgical data that may help predict postoperative complications. The AL model (ML with a variable selection of important video frames) was compared to equidistant learning for frame selection (the conventional method for frame selection from intraoperative videos based on the selection of frames at fixed time intervals). The performance metrics included F1 score, precision, and recall. Results demonstrated that AL showed a better ability to select frames for instruments and anatomy, which has the potential to reduce annotation effort while keeping ML performance high for selected features.

The substantial manual annotation effort required to build and train AI models was highlighted by de Backer et al. The authors developed an ML program to identify and annotate surgical instruments in videos to mitigate this challenge. The authors developed a bottom-up approach for team annotation of surgical instruments in robotic-assisted partial nephrectomy (RAPN) and RAMIE. This approach was developed using RAPN videos (*n* = 82) and then validated on RAMIE videos (*n* = 15). The performance metrics focused on the time per frame spent for each annotation modality (pixel and vector annotations). Their paper is a comprehensive guide to effectively developing a surgical AI program. It proposes a successful bottom-up approach for assembling annotator teams, which can be applied to any surgical annotation project. Their findings aim to lay the groundwork for AI initiatives focused on instrument detection, segmentation, and pose estimation—key areas currently posing significant challenges in the field [40].

### Surgical performance assessment

To test the feasibility of the AI prediction model for assessing surgical proficiency, Takeuchi et al. compared each AI-predicted phase duration between two groups (early and late periods) according to the learning curve for RAMIE. Certain phases, such as the duration of preparation, post-dissection to completion of the surgery, and no step phase, which were used to indicate the removal of the camera from the abdominal or thoracic cavity, were significantly shorter in the late compared to the early period. These phases are more associated with the learning curve compared to the others, which suggests that raising the team’s proficiency in introducing and changing the instruments to shorten the total duration of the surgery is essential. The performance of the AI model was assessed by comparing its predictions with a reference annotated by a surgeon. It was evaluated using normalized confusion matrices, precision, recall, *f* value, and accuracy [37]. The authors concluded that AI-based systems can be helpful in education, operating room efficiency, and evaluating surgical skills. The AI models capable of automated surgical performance and learning curve assessment can be of tremendous value for intraoperative instructions and individual feedback.

### Intraoperative guidance

As mentioned, esophageal surgery is considered one of the most technically demanding surgical procedures [26, 28]. AI-guided identification of anatomical structures could be crucial for RAMIE. The multidimensional views of the robotic setting are superior to conventional laparoscopy and open surgery. However, surgical access is different, generating a different operating viewpoint. It is, therefore, more challenging for experts and, particularly novices, to recognize the usual landmarks and anatomical structures. As such‚ AI assistance with computer-aided anatomy recognition may benefit RAMIE. One study that evaluated this was by Boer et al., who developed a deep-learning-based algorithm for anatomy recognition using thoracoscopic video frames from RAMIE for the chest component using AI [11]. The aim was to apply intelligent intraoperative surgical guidance to support surgeons in their anatomy recognition and surgical orientations to reduce morbidity from RAMIE and the learning curve associated with the procedure. The authors built a convolutional neural network (CNN) based on 1050 annotated video frames from 83 RAMIEs, using 850 frames for the training cohort and 200 frames to validate the model. The outcome measures were Dice and Hausdorff distances (primary) and pixel-wise accuracy, sensitivity, and specificity (secondary). The CNN accurately identified and predicted key anatomical structures such as the vena cava and azygous vein, aorta, and lungs to an equivalent level as an expert surgeon. Aside from the excellent predictive performance of CNN, the inference time was comparable to real-time anatomy recognition.

## Discussion

The results of this scoping review have demonstrated that the application of AI in the intraoperative video analysis of RAMIE is in its infancy, with relatively few available studies. The included AI applications were mainly focused on surgical phase recognition, surgical performance assessment, and intraoperative guidance.

The use of AI in the field of healthcare is becoming widespread. The Advanced Alert Monitor system is one example of a successful translation of AI-based technology in healthcare. It is an early detection system that alerts physicians to patients at a high risk of clinical deterioration, permitting early intervention. The use of this system significantly lowered in-hospital mortality and the need for ICU stays [41, 42]. However, the use of AI in the operating room is more novel, and at present, there are no validated applications of AI with RAMIE in the clinical setting. Outside of RAMIE, most publications regarding AI in the operating room have been in urological surgery, followed by gynecological and general surgery [43–47].

Surgical phase recognition is one of the most used AI applications in video analysis and the essential step in developing a context-aware computer-assisted surgical system [22, 36, 48, 49]. Automated surgical phase recognition is used for optimizing operating room planning, automated assistance, and intraoperative guidance [49–51]. AI applications in automated surgical phase recognition have been implemented in various MIS procedures, such as cholecystectomy, sleeve gastrectomy, and inguinal hernia repair, among others [27, 29–32, 52–54]. A study published by Eickhoff et al. reports using CV in knowledge transfer from surgical phase recognition in laparoscopic sleeve gastrectomy to the laparoscopic part of hybrid Ivor-Lewis esophagectomy [55]. Transferring the knowledge from a simpler to more complex surgical procedure could improve data efficiency and generalizability in surgical phase recognition models. In that manner, the surgical phase recognition in RAMIE, reported by Takeuchi et al., could lead to optimization of RAMIE and improve patient safety [37].

Although AI applications in automated surgical phase recognition have shown good potential and accuracy in early monocentric studies [29, 31, 32, 38, 55], several limitations exist. The primary limitations reflect the early stage of technology as developing the automated surgical phase recognition relies on users and human annotations of surgical frames, which can be resource-heavy and time-consuming. In addition, the quality of annotations can vary, and manual annotation is time-consuming and prone to human error, impacting the model’s performance. Furthermore, the program depends on high-quality multicentric annotated surgical videos to avoid biases in the surgical phase. These are often scarce due to privacy concerns, variability in surgical techniques, and limited availability. Surgeons rely on their training and experiences to guide their decision-making in esophageal surgery. However, AI models have the potential to aid in objective surgical decision-making, providing empirically supported evidence. Although not associated with RAMIE, Kumazu et al. developed an AI-based algorithm to identify loose connective tissue fibers to define safe dissection planes in robotic-assisted gastrectomy to support surgical decision-making [56]. The application of this technology may be beneficial in patients with previous abdominal surgery generating adhesions or when it is challenging to identify anatomical planes. AI in esophageal surgery has focused mainly on intraoperative structure detection, such as real-time detection of the recurrent laryngeal nerve [57]. In RAMIE, Brandenburg et al. demonstrated that AI can be used in the intraoperative detection of video frames with relevant bleeding to predict overall patient outcomes [39]. Such AI intraoperative surgical guidance can improve RAMIE safety and surgical quality.

One of the most promising applications of intraoperative video analysis is improving surgical training and providing automated and real-time feedback to surgeons [58]. The learning curve for RAMIE to proficiency varies in the current literature from 20 to 70 procedures [59–61]. Strategies to minimize the learning curve have been undertaken by Fuchs et al., who developed a 6-step modular program for RAMIE. The program deconstructed the RAMIE into smaller modular phases to simplify the tasks. This proved highly effective for the safe introduction of RAMIE to expert and trainee surgeons alike [62]. Beyond RAMIE, various surgical procedures have explored AI-driven intraoperative video analysis. A narrative review examining AI applications in laparoscopic cholecystectomy, based on 32 studies including randomized controllet trials, systematic reviews, and meta-analyses, highlighted AI’s potential to enhance safety, improve surgical training, and reduce bile duct injury risk by refining anatomical structure detection [63]. Similarly, AI’s role in urological surgery has been assessed, with a recent review evaluating its use in robotic prostatectomy. The authors concluded that AI serves as an adjunct to support the surgeon and surgical team in optimizing patient outcomes rather than replacing them. This underscores the early stage of AI integration in surgery and its current limitations, including a lack of validated clinical models, emphasizing that AI remains a complement to human expertise rather than a substitute [64].

A particular strength of the AI application in intraoperative video analysis that has not been evaluated in RAMIE to date is surgical quality assessment (SQA). Currently, SQA evaluation relies on surgeon credentialing, auditing of surgical notes and intraoperative reports, data monitoring, pathology review, and intraoperative manual video assessment by surgeons, which is subject to bias and extremely time-consuming [65]. Using AI in intraoperative video analysis in MIS procedures allows the unique opportunity to assess the quality of the surgery and benchmark the procedure. The standardized procedure using SQA with AI has tremendous potential to generate benefits for academic purposes and deliver high-quality surgery.

AI-based research in surgery is a relatively new field, so methodological standards are still being developed or established in the scientific and surgical communities. Reporting guidelines specific to research and innovation in this field are created to help produce interpretable, reproducible, and scalable scientific work. Researchers may struggle with reporting their methodology, data collection, training, and testing of AI algorithms. Similarly, journal editors and peer-reviewers may have difficulty critically appraising manuscripts to determine if their readership can generalize or interpret the findings (mostly surgeons who lack a technical background in this field). These issues have been partly addressed by developing guidelines or modifying existing guidelines specific to AI, such as STARD-AI, where the main aim is to help critical stakeholders appraise the quality and compare the diagnostic test accuracy of AI models [34], CLAIM (Checklist for Artificial Intelligence in Medical Imaging), CONSORT-AI (Consolidated Standards of Reporting Trials-AI), SPIRIT-AI (Standard Protocol Items: Recommendations for Interventional Trials-AI), FUTURE-AI (Fairness Universality Traceability Usability Robustness Explainability-AI), MI-CLAIM (Minimum Information about Clinical Artificial Intelligence Modeling), MINIMAR (Minimum Information for Medical AI Reporting), and RQS (Radiomics Quality Score) [66].

This overview focused on RAMIE and did not include the laparoscopic approach in esophageal surgery. Although both RAMIE and laparoscopic esophagectomy provide videos that can be used in AI research and have similar surgical phases, there are some differences in the data they provide for AI research. One of the differences is the quality of the video. For example, RAMIE videos can have higher quality images and fewer interferences due to stable camera position. Instrument movement can differ due to more advanced dexterity and ergonomic optimization. Therefore, it is essential not only to harvest the benefits of the similarities of the two surgical approaches in AI research but also to emphasize the differences to optimize the AI applications in different surgical settings.

Ethical considerations in AI applications for surgery are crucial, particularly regarding transparency, validation, and accountability. AI models frequently function as “black boxes,” hindering the validation of their internal decisions and raising concerns about potential biases and misleading outputs. An overreliance on AI without sufficient human oversight could pose significant risks in surgical decision-making. Therefore, it is essential to establish rigorous validation protocols and implement regulatory frameworks to ensure that AI-driven decisions are both reliable and safe. The included studies validated their AI algorithms through cross-validation with an independent dataset and comparison with the expert annotated dataset, showing the reliability of the AI algorithms. However, to fully establish the validity and reliability of the AI algorithm, validation through an inter-rater agreement among a team of surgical experts is necessary, especially in clinical applications.

The use of AI in RAMIE is in its infancy but it is a growing and promising field. Scientific research on this topic is expected to grow substantially over the next decade, especially since standardized AI study reporting methods are being developed. These methods enable direct assessment and comparison between studies and achieve transparency in the scientific reporting of AI-based evidence. Acquiring high-quality research on this topic is crucial to address the current shortage of validated clinical tools for AI-driven surgical video analysis, ensuring their safety and effectiveness before widespread clinical adoption.

Currently, AI-based autonomy of robotic systems is a long-term endeavor that necessitates large-scale validated studies to build confidence and demonstrate effectiveness prior to adoption in the clinical setting; however, considering the rapid rate of sophisticated data acquisition and the successful translation of AI-based technologies in other aspects of medicine, this may be a possibility in the near future.

## Conclusion

Overall, the application of AI in RAMIE is expected to provide substantial improvements in the assessment of surgical skills, standardization of techniques, and improvement of the intraoperative phase of this surgery. Currently, the most common applications in intraoperative video analysis are surgical phase recognition, identification of anatomical landmarks, and intraoperative guidance. Soon, one of AI’s most valuable applications will be surgical quality assessment of MIS procedures. By leveraging AI, surgeons may achieve higher levels of consistency and accuracy, ultimately leading to improved clinical outcomes and more efficient surgical processes. Despite the current limitations, the potential benefits make AI’s continued development and integration in surgical practice an exciting and worthwhile endeavor.

## Supplementary Information

Below is the link to the electronic supplementary material.

Supplementary file1 (DOCX 14 KB)
