# Supplementary material for: Artificial intelligence for intraoperative video analysis in robotic-assisted esophagectomy
Source: Surg Endosc. 2025 Mar 31;39(5):2774–83. doi: 10.1007/s00464-025-11685-6 (PMC12041040; doi:10.1007/s00464-025-11685-6)
Supplement: Supplementary file 1 — Supplementary file1 (DOCX 14 KB) [file 464_2025_11685_MOESM1_ESM.docx]

**Supplementary file 1**

Embase Classic+Embase <1946 to 14 August 2023>

1 machine learning.mp. or exp machine learning/ 538694

2 artificial intelligence.mp. or exp artificial intelligence/ 129545

3 algorithm.mp. or exp learning algorithm/ or exp algorithm/ 790020

4 deep learning.mp. or exp deep learning/ 89950

5 exp large language model/ or exp natural language processing/ or large language.mp. 19519

6 1 or 2 or 3 or 4 or 5 1134679

7 exp robot assisted surgery/ or exp robot/ or robot.mp. 75278

8 exp esophagectomy/ or exp minimally invasive esophagectomy/ or esophagectomy.mp. 21924

9 oesophagectomy.mp. 3326

10 8 or 9 24613

11 7 and 10 840

12 6 and 11 117
